# Mg(OH)_2_‐Facilitated Liquid‐Phase Conversion of Lactic Acid into 1,2‐Propanediol over Cu: An Experimental and Theoretical Study

**DOI:** 10.1002/cssc.201902347

**Published:** 2019-10-21

**Authors:** Xinde Wang, Anna Katharina Beine, Peter J. C. Hausoul, Regina Palkovits

**Affiliations:** ^1^ Institut für Technische und Makromolekulare Chemie RWTH Aachen University Worringerweg 2 52074 Aachen Germany

**Keywords:** alcohols, carboxylic acids, copper, hydrogenolysis, magnesium

## Abstract

Mg(OH)_2_ is found to exhibit superior performance in the liquid‐phase conversion of lactic acid (LA) into 1,2‐propanediol over Cu. A conversion of 90 % with a selectivity of 98 % is achieved at 513 K and 5 MPa H_2_. Mg(LA)_2_ could be identified as a crucial intermediate in this reaction, as it undergoes faster conversion than the combination of LA and Mg(OH_2_) and regeneration of Mg(OH)_2_ through the conversion of Mg(LA)_2_ as a substrate. DFT calculations reveal that the energetic span of the reaction decreases from 46.6 kcal mol^−1^ catalyzed with no cation to 43.6 kcal mol^−1^ with [Mg(OH)]^+^, confirming the facilitating effect of Mg(OH)_2_.

## Graphical abstract

**Milk and alcohol**: Mg(OH)_2_ exhibits superior performance in the liquid‐phase conversion of lactic acid (LA) into 1,2‐propanediol over Cu. A conversion of 90 % with a selectivity of 98 % is achieved at 513 K and 5 MPa H_2_. Mg(LA)_2_ is found to be a crucial intermediate in this reaction. DFT calculations reveal that the energetic span of the reaction decreases in the presence of [Mg(OH)]^+^.

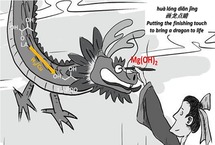

Lactic acid (LA) has attracted increasing attention, owing to its use as monomer for biodegradable polylactic acid (PLA).^1^ Consequently the production of l‐lactic acid, a naturally occurring organic acid, by bacterial fermentation of carbohydrates has been steadily rising.^2^ Although relatively selective, still significant amounts of the other enantiomers (e.g., d‐lactic acid) are produced, causing extra expense for separation for the production of PLA. LA is also produced as an unavoidable side product in the hydrogenolysis of biomass to glycols.^3^ However, in this case LA is racemic and generally unsuitable for the production of PLA. Hence routes for the conversion of LA into value‐added products are important when considering its large‐scale production.^4^ Particularly, the direct conversion of biogenic LA into 1,2‐propanediol (1,2‐PDO) in the liquid phase is attractive, given the potential for CO_2_ reduction and the wide application of 1,2‐PDO as a monomer and as a constituent in antifreezes (Scheme 1).^5^

**Scheme 1:**
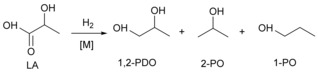
Metal‐catalyzed hydrogenolysis of LA to 1,2‐PDO and alcohols.

Hydrogenation of LA was initially reported by Broadbent and Whittle, who obtained a yield of 84 % at 423 K and 26 MPa H_2_ using a Re‐black catalyst.^6^ Zhang et al. investigated multiple catalysts and observed superior activity for Ru/C.^7^ A conversion of 95 % with 90 % selectivity to 1,2‐PDO was obtained at 423 K and 14.5 MPa H_2_. Furthermore, Takeda et al. improved the catalytic performance of Ru/C by doping with MoO_*x*_ species, which led to a yield of 95 % at 393 K and 8 MPa H_2_.^8^ Besides highly active Ru catalysts, non‐noble metals have also been investigated.^9^ Cortright et al. reported full conversion with a selectivity of 88 % to 1,2‐PDO over Cu/SiO_2_ at 473 K in the gas phase.9g The authors proposed a two‐step reaction mechanism involving dehydration over the SiO_2_ support and hydrogenation over Cu. Notably, in the liquid phase reaction no conversion of LA was observed. However, in our previous study on the hydrogenolysis of sorbitol in water we observed that conversion of LA into 1,2‐PDO leads to increasing glycol selectivity.^10^ Herein we present the direct conversion of LA under basic conditions by using SiO_2_‐supported Cu catalysts. The influence of the added base on the course of the reaction and the reaction mechanism was studied in detail. DFT calculations were performed and suggest a strong influence of the cation on the reaction mechanism.

The Cu/SiO_2_ catalyst was prepared by using the ammonia evaporation precipitation method. The metal loading was determined by inductively coupled plasma optical emission spectroscopy (ICP‐OES) to be 24.2 wt %. N_2_ physisorption revealed that Cu/SiO_2_ had a surface area of 279 m^2^ g^−1^ and a pore volume of 0.71 cm^3^ g^−1^. The addition of Cu did not modify the surface area (287 m^2^ g^−1^) or pore volume (0.88 cm^3^ g^−1^) of SiO_2_ significantly. The adsorption and desorption isotherms are shown in Figure S1 (see the Supporting Information). The particle distribution was investigated by using TEM (Figure 1). The Cu particle size is 13.1±3.6 nm.

**Figure 1:**
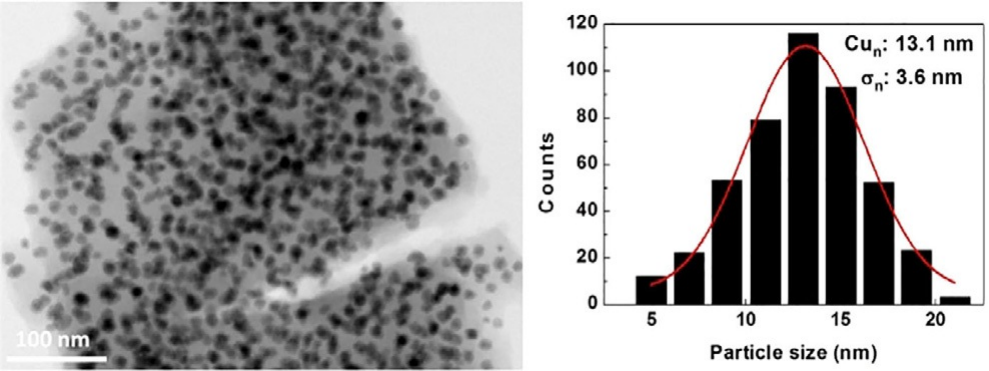
TEM image and particle size distribution of Cu/SiO_2_.

The influence of temperature on the conversion was investigated in the presence of Mg(OH)_2_ at 5 MPa H_2_ (Figure 2 a). High selectivities of up to 98 % were achieved in all cases, indicating that Cu is an excellent catalyst to produce 1,2‐PDO. The conversion increases from 4 % at 453 K to 63 % at 513 K. Notably, a conversion of 40 % was obtained at 473 K, confirming that Cu can also be employed at lower temperatures. The increased reaction rate is partially attributed to the higher solubility of Mg(OH)_2_ at the higher temperature, favoring the generation of Mg(LA)_2_. Furthermore, the influence of the H_2_ pressure on the reaction rate was investigated (Figure S2). At 513 K, the conversion increased from 16 % at 1 MPa to 86 % at 4 MPa, although further increase in the H_2_ pressure did not lead to higher conversion. The pressure dependence below 4 MPa is attributed to mass transfer limitations, owing to the three‐phase reaction. To exclude this effect, all reactions were carried out at 5 MPa H_2_. To test the long‐term catalyst stability, five consecutive recycling experiments were carried out using 25 wt % Cu/SiO_2_ (Figure 2 b). Conversion continuously decreased from 97 % in the first run to 23 % in the fifth run. ICP‐OES of the reaction solution showed only minimal Cu leaching (≤0.1 %; Table S1). TEM of the spent catalyst revealed a significant agglomeration of the nanoparticles (Figure 2 c). The Cu particle size increased from 13.1 nm for the fresh catalyst to 57.8 nm for the five‐times‐recycled one. This is in line with our previous observations on the conversion of sorbitol over Cu/AC, where the observed deactivation could be attributed to sintering of nanoparticles.^10^ This once again demonstrates that further studies on the synthesis of stable Cu catalysts are necessary.

**Figure 2:**
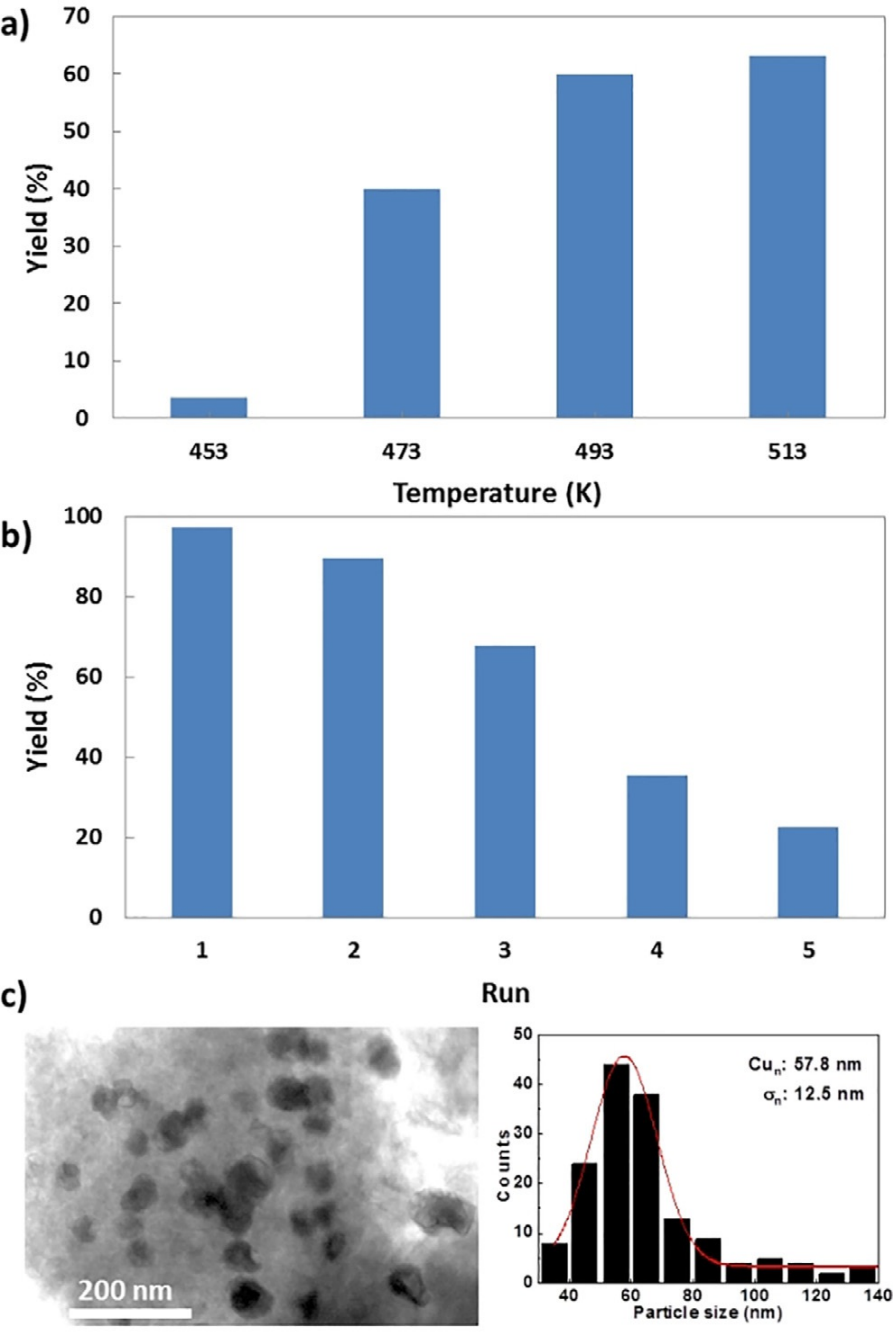
a) Temperature dependence of LA conversion over 25 % wt% Cu/SiO_2_. Reaction conditions: catalyst (0.1 g), LA (0.5 g), Mg(OH)_2_ (0.4 g), H_2_O (20 mL), H_2_ (5 MPa), 4 h. b) Recycling of 25 % Cu/SiO_2_ over five runs. Reaction conditions: 25 % Cu/SiO_2_ (0.1 g), Mg(LA)_2_ (0.56 g), H_2_O (20 mL), 513 K, H_2_ ()5 MPa, 4 h. c) TEM image and particle distribution of the five‐times‐recycled 25 % Cu/SiO_2_ catalyst.

The influence of the type of base on the conversion of LA over Cu/SiO_2_ is displayed in Figure 3 a. In every experiment, the amount of OH^−^ in the reaction mixture was kept the same. A reaction without base gave almost no conversion, indicating that Cu/SiO_2_ alone is inactive, which is in accordance with the results reported by Cortright et al.9g The bases NaOH, Sr(OH)_2_ and La(OH)_3_ gave almost no conversion (<3 %). KOH, Zr(OH)_2_ and Ca(OH)_2_ reached slightly higher conversion of up to 10 %. However, with Mg(OH)_2_ a significant increase in activity was achieved. 63 % of LA was converted within 4 h and extending the reaction time to 24 h led to 90 % conversion (Figure 3). When the reaction was carried out in the presence of Mg(OH)_2_ but without Cu/SiO_2_, conversion again did not exceed 3 %. To our knowledge, this is the first example of the conversion of LA into 1,2‐PDO with promising yield over a Cu catalyst in the liquid phase. Increasing the equivalence of Mg(OH)_2_ (see Figure S3) shows that the reaction is accelerated. This suggests that the dissolution of Mg(OH)_2_ is rate‐limiting. To test whether solid Mg(OH)_2_ participates in the reaction, a test was performed with magnesium lactate (Mg(LA)_2_), which is fully water‐soluble. The time course of the reaction is displayed in Figure 3 b. Compared to the reaction of LA in the presence of Mg(OH)_2_, the conversion of Mg(LA)_2_ proceeds much faster. A conversion of up to 92 % was obtained in the first 2 h, whereas for LA+Mg(OH)_2_ only 42 % conversion was observed. Given the poor solubility of Mg(OH)_2_, the greatly accelerated conversion can be attributed to the high solubility of Mg(LA)_2_. The evolution of the Mg^2+^ concentration during the reaction was analyzed by ICP‐OES. The concentration of Mg^2+^ decreased from 73.9 mmol L^−1^ at 0.5 h to 6.6 mmol L^−1^ at 2 h (Figure 3 b). After the reaction, the pH of the solution was around 10, which is higher than would be expected from Mg(LA)_2_ alone. Furthermore, the formation of a white powder that could be dissolved in HCl was observed after the reaction. This confirms that Mg(OH)_2_ was generated during the reaction. These observations suggest that soluble Mg species play an important role in the conversion of LA facilitated by Mg(OH)_2_.

**Figure 3:**
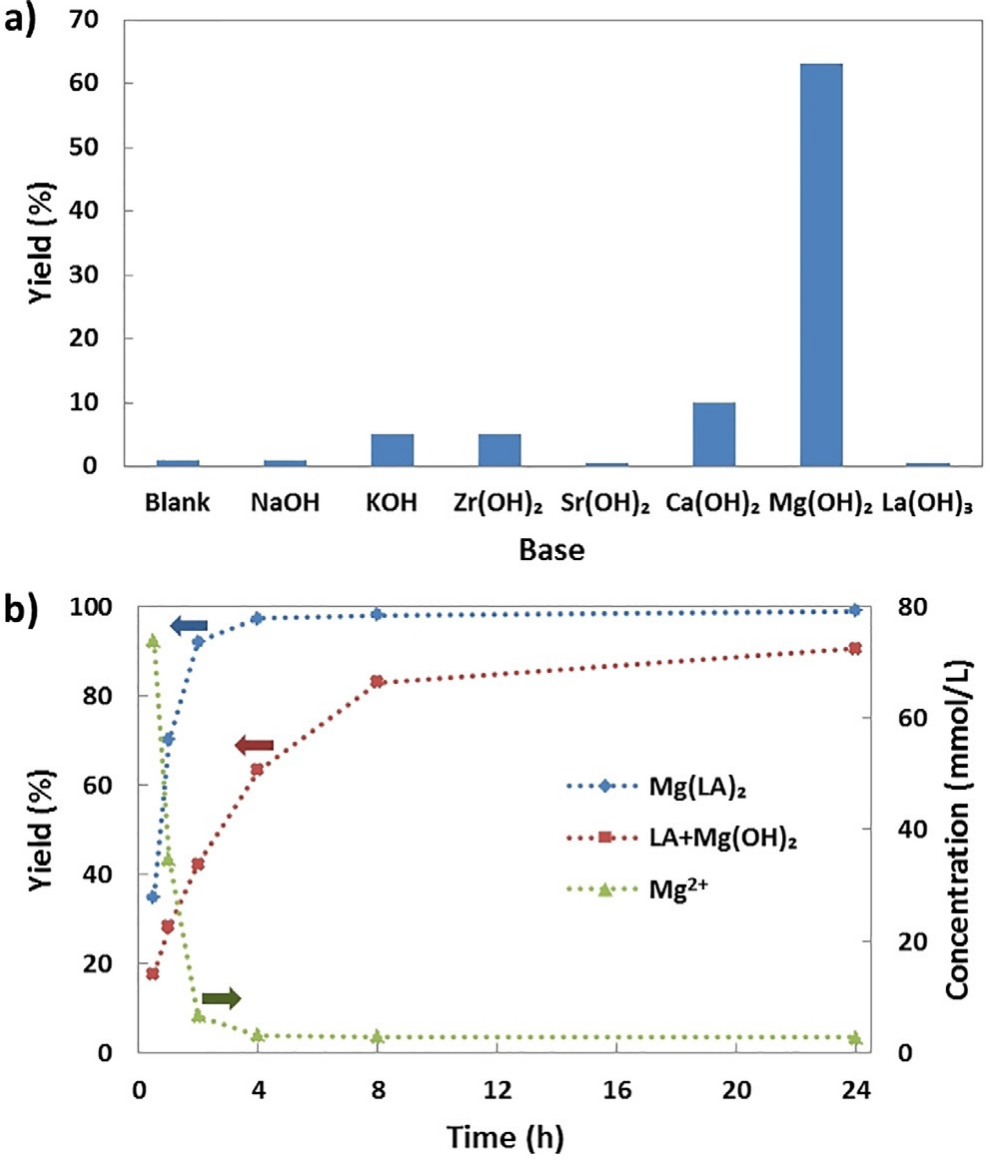
a) Influence of base on LA conversion over 25 % Cu/SiO_2_. Reaction conditions: 25 % Cu/SiO_2_ (0.1 g), LA (0.5 g), OH^−^ (68.5 mmol), H_2_O (20 mL), 513 K, H_2_ (5 MPa), 4 h. b) Conversion of Mg(LA)_2_ and LA in the presence of Mg(OH)_2_ over Cu/SiO_2_ and the evolution of Mg^2+^ concentration over time. Reaction conditions: 25 % Cu/SiO_2_ (0.1 g), Mg(LA)_2_ (0.56 g), LA (0.5 g), Mg(OH)_2_ (0.4 g), H_2_O (20 mL), 513 K, H_2_ (5 MPa).

To obtain deeper insights into the role of Mg^2+^ on the conversion of lactate to 1,2‐PDO, DFT calculations at the B3LYP/6‐311++G**/SMD(water) level of theory were carried out.^11^ The proposed reaction mechanisms and their energy profiles are displayed in Figure 4 (see Figure S4 for the calculated geometries and Table S2 for obtained *d*G values). As both Cu and Mg(OH)_2_ are necessary for the conversion, a synergistic reaction mechanism composed of a base/cation‐catalyzed part and a Cu‐catalyzed part is proposed. The OH^−^ catalyzed reaction sequence [i.e. with no cation (NC)] is considered as a benchmark. Preliminary calculations showed that dissolved Mg(OH)_2_ is hydrated and in equilibrium with the cationic species [Mg(OH)(H_2_O)_5_]^+^ and [Mg(H_2_O)_5_]^2+^ and OH^−^.^12^ In the presence of LA, OH^−^ is neutralized, leading to the formation of LA^−^ and after coordination with Mg to [Mg(OH)(LA)(H_2_O)_2_)] and [Mg(LA)(H_2_O)_3_]^+^, respectively (**IM1**). Neutralization is strongly exothermic [−23.6 (NC), −27.2 ([Mg(OH)]^+^) and −27.1 (Mg^2+^) kcal mol^−1^]. The lower *d*G of the magnesium lactate complexes suggests that magnesium cations have a high affinity for the lactate anion. Next, a proton transfer from the α‐hydroxy group to the carboxylic group leads to **IM2** and activation of the α‐carbon. In all cases proton transfer are endothermic, requiring 23.6 (NC), 15.3 ([Mg(OH)(LA)(H_2_O)_2_]) and 14.9 kcal mol^−1^ ([Mg(LA)(H_2_O)_3_]^+^), respectively. For path NC, the proton is bridged between the hydroxy and carboxylic acid groups, whereas for the Mg‐ion paths, the proton is fully shifted to the carboxylic acid. Compared to NC, binding with cation is highly beneficial and lowers the energy for proton transfer by 8 kcal mol^−1^. Subsequently, a hydride shifts from the activated α‐carbon to the carbonyl via transition state **TS1** and results in the formation of gem‐diolate **IM3**. For this step, the Mg‐ion paths exhibit higher barriers of 29.9 ([Mg(OH)]^+^) and 28.3 (Mg^2+^) kcal mol^−1^ as compared to 23.0 for NC. The hydride is approximately located over the middle of C−C bond.

**Figure 4:**
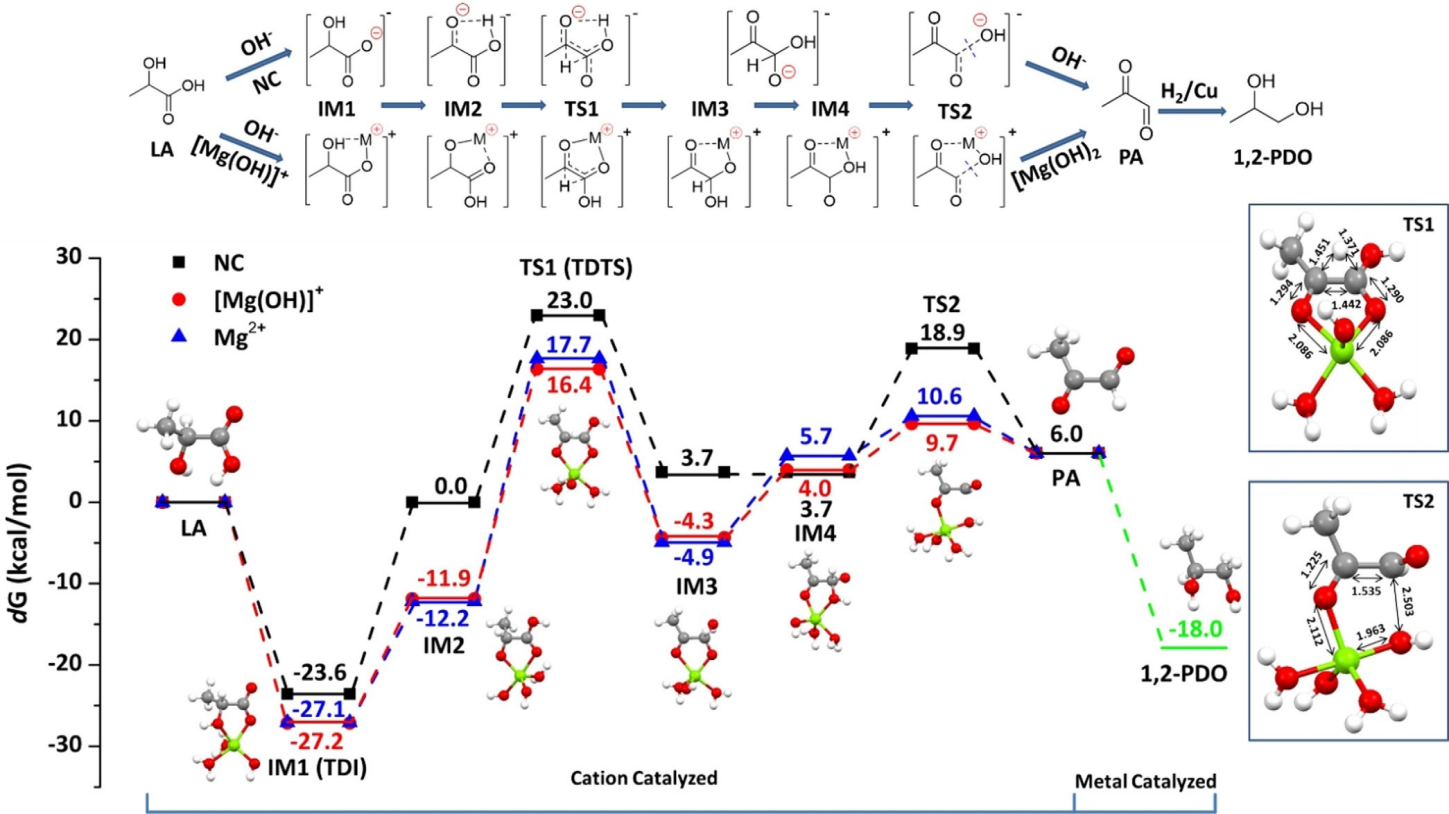
Energy profiles for the conversion of LA into 1,2‐PDO in the presence of base conditions (OH^−^) with no cation (NC), [Mg(LA)]^+^ and Mg^2+^ coupled with metal catalyst. Gibbs free energy values are calculated in water and given in kcal mol^−1^. All atomic distances are given in Å (SMD/B3LYP‐D3/6‐311++G**); H (white), C (grey), O (red) and Mg (green). TDI and TDTS represent TOF‐determining intermediate and TOF‐determining transition state.

For path NC, elimination of OH^−^, going through **TS2** and yielding pyruvaldehyde (PA), can proceed directly. In contrast, for the Mg‐ion paths, a proton transfer from one hydroxy group of the gem‐diol to the Mg‐bound hydroxy (**IM4**) occurs before elimination of OH^−^ (via **TS2**). As such the base is regenerated at the end of the reaction, which is in accordance with our observations. Finally, PA is hydrogenated over Cu to give 1,2‐PDO.

To evaluate the catalytic efficiency of each route, the energetic span (*δE*) model, which corresponds to the apparent activation energy of the entire catalytic cycle, is applied.^13^ Generally, *δE* is determined based on the TOF‐determining transition state (TDTS) and TOF‐determining intermediate (TDI). As it has the lowest *d*G among all investigated species, **IM1** is assigned to the TDI. As the *d*G of **TS1** is higher than that of **TS2**, **TS1** is assigned to TDTS. Moreover, the TDI appears before the TDTS, and *δE* is determined by *d*G_**TS1**_−*d*G_**IM1**_. As such, the *δE* for the NC, [Mg(OH)]^+^‐, and Mg^2+^‐facilitated catalytic routes are 46.6, 43.6, and 44.8 kcal mol^−1^, respectively. Although *δE* values of [Mg(OH)]^+^ and Mg^2+^ are different, both are significantly lower than that of NC.

The Gibbs energy profiles of the conversion of LA into 1,2‐PDO involving the rate‐determining states in the presence of NC, Ca^2+^ and Mg^2+^ are shown in Figure 5. The *d*G of TDI and TDTS for Ca^2+^ are −25.5 and 21.4 kcal mol^−1^, respectively. The *δE*(Ca^2+^) is comparable with *δE*(NC), and 2.1 kcal mol^−1^ higher than *δE*(Mg^2+^). Therefore, the obtained DFT result is in well accordance with the experimental observations that Mg(OH)_2_ facilitates significantly the conversion of LA in water.

**Figure 5:**
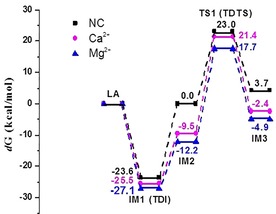
Energy profiles for the conversion of LA into 1,2‐PDO involving rate‐determining states in the presence of NC, Ca^2+^, and Mg^2+^. Gibbs free energy values are calculated in water and given in kcal mol^−1^.

In summary, we have shown that the liquid‐phase conversion of LA into 1,2‐PDO can be efficiently achieved with promising yield by using a Cu catalyst in the presence of base. Among various bases, Mg(OH)_2_ was the most active. Mg(LA)_2_ was experimentally identified as the key intermediate. DFT calculations demonstrated that the *δE* value of the reaction in the presence of Mg cations is decreased by 3 kcal mol^−1^ compared to that without cations, confirming the facilitating effect. This combined study provides a promising direction to take advantage of the side product LA in biomass conversion into glycols and deeper insights into this new route.

## Conflict of interest

*The authors declare no conflict of interest*.

## Supporting information

As a service to our authors and readers, this journal provides supporting information supplied by the authors. Such materials are peer reviewed and may be re‐organized for online delivery, but are not copy‐edited or typeset. Technical support issues arising from supporting information (other than missing files) should be addressed to the authors.

Supplementary
