# Supplementary material for: Mg(OH)2‐Facilitated Liquid‐Phase Conversion of Lactic Acid into 1,2‐Propanediol over Cu: An Experimental and Theoretical Study
Source: ChemSusChem. 2019 Oct 21;13(1):126–30. doi: 10.1002/cssc.201902347 (PMC6973064; doi:10.1002/cssc.201902347)
Supplement: Supplementary file 1 — Supplementary [file CSSC-13-126-s001.pdf]

CHEMISTRY & SUSTAINABILITY
CHEMSUSCHEM
ENERGY & MATERIALS

Supporting Information

$Mg(OH)_2$-Facilitated Liquid-Phase Conversion of Lactic Acid into 1,2-Propanediol over Cu: An Experimental and Theoretical Study

Xinde Wang, Anna Katharina Beine, Peter J. C. Hausoul, and Regina Palkovits*[a]

cssc_201902347_sm_miscellaneous_information.pdf

$Mg(OH)_2$ facilitated liquid-phase conversion of lactic acid to 1,2-propanediol over Cu– an experimental and theoretical study

*Xinde Wang, Anna Katharina Beine, Peter J. C. Hausoul and Regina Palkovits**

Institut für Technische und Makromolekulare Chemie, RWTH Aachen University, Worringerweg 2, 52074 Aachen, Germany.

Email: palkovits@itmc.rwth-aachen.de

EXPERIMENTAL

All chemicals were used as received. $Cu(NO_3)_2{\cdot}3H_2O$ ($\geqslant$ 98%), $Mg(OH)_2$ (98%), $Ca(OH)_2$ ($\geqslant$ 95%), KOH (90%), NaOH (97%), $Zr(OH)_2$ (97%), $Sr(OH)_2$ (94%), $La(OH)_3$ (99.9%), 1,2-PDO (99%), $Mg(LA)_2$ ($\geqslant$ 95%), were obtained from Sigma Aldrich. Silica gel (40%) was obtained from ACROS Organics. Lactic acid (90%) was obtained from Fluka.

Catalyst Preparation

Cu/SiO_2 catalyst was prepared *via* an ammonia evaporation precipitation method. 4.05g $Cu(NO_3)_2{\cdot}3H_2O$ was dissolved in deionized water (25 mL) and stirred for 0.5 h in a round bottom flask. 10.7 ml $NH_{3(aq)}$ solution (25 wt. %) was diluted to 50 ml using deionized water. Afterwards, the diluted $NH3_{(aq.)}$ was dropwise added into Cu solution. 1 h after the complete addition of $NH_{3(aq)}$, 40 wt. % colloidal silica was added to the slurry. The mixture was stirred at 750 rpm for overnight at room temperature in order to obtain a well-dispersed suspension. The ammonia was removed by heating in an oil bath at 363 K until the pH of the slurry was 7. The resulting powder was filtered of and washed with 2 L deionized water and subsequently dried in a drying oven at 353 K for overnight. The prepared materials were calcined at 823 K for 4 h. The activation took place under H_2 at 723 K with 5 K/min for 4 h. The pristine catalysts were stored under Ar atmosphere.

Catalyst Characterization

N_2-physisorption analysis. The textual properties of the samples were determined by N_2-physisorption at 77 K using a QUADRASORB SI automated surface area and pore size analyzer. Samples were pre-treated in vacuum at 473 K for overnight. The pore-size distribution was estimated by the Barrett-Joyner-Halenda (BJH) method from the desorption branches of the adsorption isotherms. Specific surface areas were calculated using the Brunauer−Emmett−Teller (BET) method.

Transmission electron microscopy (TEM). Images were obtained using a Philips TECNAI G2 F20 system electron microscope at 100 kV equipped with a field emission gun. The sample powder was dispersed in isopropanol by ultra-sonication. Drops of the suspension were applied onto a copper grid-supported transparent carbon foil and dried in air. The particle size was determined by using the software ImageJ.

Inductively Coupled Plasma Optical Emission Spectroscopy (ICP-OES). The catalyst was digested using a combination of the concentrated nitric acid and hydrochloric acid, followed by measurement of the solution by ICP-OES performed on an ICP Spectroflame D by Spectro.

Catalyst Activity Tests

Hydrogenolysis of LA was carried out in a 50 mL stainless steel autoclave equipped with a Teflon inlet. In a typical run, aqueous LA solution (0.5 g LA, 20 g H_2O), $Mg(OH)_2$ (0.4 g) and 0.1 g 25%Cu/SiO_2 catalyst were charged into the autoclave. The reactor was purged five times and pressurized to 5 MPa with H_2. The sealed autoclave was heated to 513 K and stirred at a speed of 750 rpm. Samples were taken periodically.

Recycling Tests

Recycling tests were carried out with 25%Cu/SiO_2 catalyst. After each test, the catalyst was washed by deionized water. 5% fresh catalyst was charged in next run in order to supplement catalyst loss during the recycling.

Analytical Method

The gas-phase was collected using a gasbag and analyzed by GC performed on a Agilent HP 6890 instrument with a Shin Carbon ST micropacked GC column (308-373 K, TCD, He). Liquid-phase products were analyzed by HPLC performed on a Shimadzu system (Rezex ROA Organic Acid H^+ (8%) column by Phenomenex, eluent: 0.05 M H_2SO_4, the mobile phase flow rate is 0.6 ml/min with a column temperature of 343 K). The LA conversion (Eq. 1) and selectivity (Eq. 2) were calculated on the basis of carbon.

$$Conversion = \frac{mole\ of\ reactant\ charged - mole\ of\ reactant\ left}{mole\ of\ reactant\ charged} \times 100\% \quad \text{(Eq. 1)}$$

$$Selectivity = \frac{mole\ of\ product \times C\ atoms\ in\ product}{(mole\ of\ reactant\ charged - mole\ of\ reactant\ left) \times C\ atoms\ in\ reactant} \times 100\% \quad \text{(Eq.2)}$$

DFT Calculation

All of the calculations were performed with the NWCHEM software package based on the DFT method.[1] The geometries of all of the transition states, reactants and intermediates involved in the reaction were fully optimized using a hydrated cluster in conjunction with the continuum solvation model of SMD at the B3LYP/6-311++G** level of theory with dispersion correction vdw 3. Harmonic frequency calculations were performed at the equilibrium geometries to confirm first-order saddle points and local minima on the potential energy surfaces, and to estimate the zero-point energy, as well as the thermal and entropic corrections at 298.15 K and 1 atm. The correlation between the stable structures and the transition states was verified by analyzing the corresponding imaginary frequency mode. For estimation of the Gibbs free energy, direct calculations in combination with frequency analysis in solution were performed.

Energetic span *δE* is obtained on the basis of TOF-determining intermediate (TDI) and transition state (TDTS) (Eq. 3).[2]

$$\delta E = G_{TDTS} - G_{TDI} \quad \text{(Eq. 3)}$$

Results and Discussion

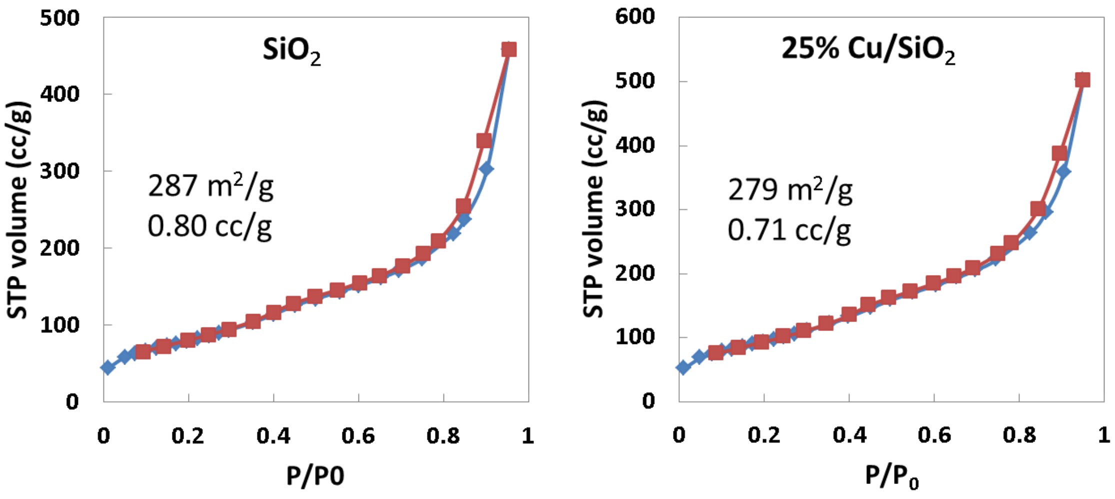

Figure S1. Nitrogen adsorption/desorption isotherms and corresponding specific surface area and pore volume of SiO_2 and the supported Cu catalyst.

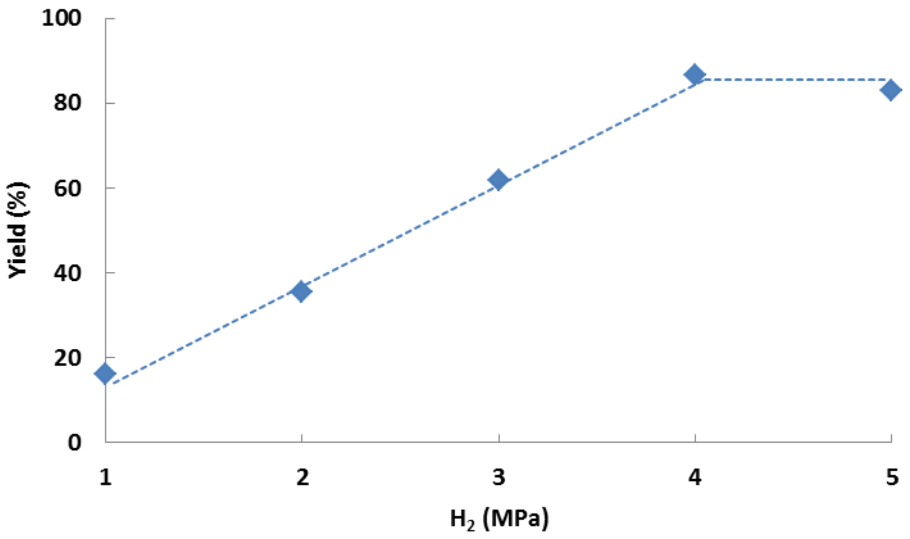

Figure S2. Conversion of LA for varying H_2 pressure. Reaction conditions: 0.2 g 25%Cu/SiO_2, 0.5 g LA, 0. 4g $Mg(OH)_2$, 20 ml H_2O, 513 K, 4 h.

Table S1. Cu leaching in each run. Reaction conditions: 0.1g 25%Cu/SiO_2, 0.56g $Mg(LA)_2$, 20ml H_2O, 513 K, 5 MPa H_2, 4h.

Run	Cu leaching (%)
1	0.11
2	0.09
3	0.08
4	0.04
5	0.08

Table S2. *Δ*G of each species.

Species	OH^-				$Mg(OH)_2$				$[Mg(OH)]^+$				Neutral			
	$E_{sol.}$	S	H	ΔG	$E_{sol.}$	S	H	ΔG	$E_{sol.}$	S	H	ΔG	$E_{sol.}$	S	H	ΔG
IM1	-343.31	55.60	78.22	-343.26	-772.27	103.05	120.46	-772.16	-772.73	111.51	119.26	-772.61	-	-	-	-
IM2	-343.28	55.70	76.71	-343.22	-772.24	102.4	120.32	-772.13	-772.71	111.62	119.74	-772.59	-	-	-	-
TS1	-343.23	52.01	76.78	-343.19	-772.19	99.47	119.85	-772.09	-772.66	107.61	115.19	-772.54	-	-	-	-
IM3	-343.27	54.91	80.18	-343.22	-772.23	101.2	122.23	-772.12	-772.69	110.82	126.35	-772.58	-	-	-	-
IM4	-	-	-	-	-772.21	101.27	119.62	-772.11	-772.68	111.22	117.34	-772.56	-	-	-	-
TS2	-	-	-	-	-772.22	101.94	124.56	-772.12	-772.68	111.21	121.61	-772.56	-	-	-	-
PVD	-267.25	44.88	74.57	-267.21	-772.20	101.36	129.72	-772.1	-772.67	109.33	129.96	-772.56	-	-	-	-
OH^-	-75.99	7.51	41.14	-76.00	-	-	-	-	-	-	-	-	-	-	-	-
$Mg(OH)_2$	-	-	-	-	-581.43	70.97	110.76	-581.37	-	-	-	-	-	-	-	-
$[Mg(OH)]^+$	-	-	-	-	-	-	-	-	-581.90	81.64	104.79	-581.82	-	-	-	-
LA	-	-	-	-	-	-	-	-	-	-	-	-	-343.76	64.31	78.05	-343.69
1,2-PDO	-	-	-	-	-	-	-	-	-	-	-	-	-269.69	75.62	76.07	-269.01
H_2O	-	-	-	-	-	-	-	-	-	-	-	-	-76.47	15.54	46.47	-76.47

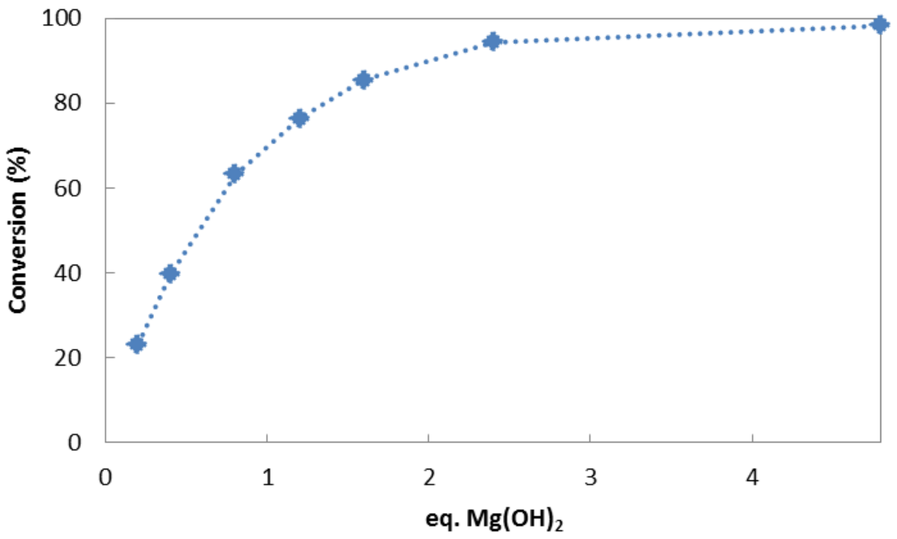

Figure S3. The effect of the eq. $Mg(OH)_2$ on the reaction. Reaction conditions: 0.1 g 25%Cu/SiO_2, 0.5 g LA, 20 ml H_2O, 513 K, 5 MPa H_2, 4 h.

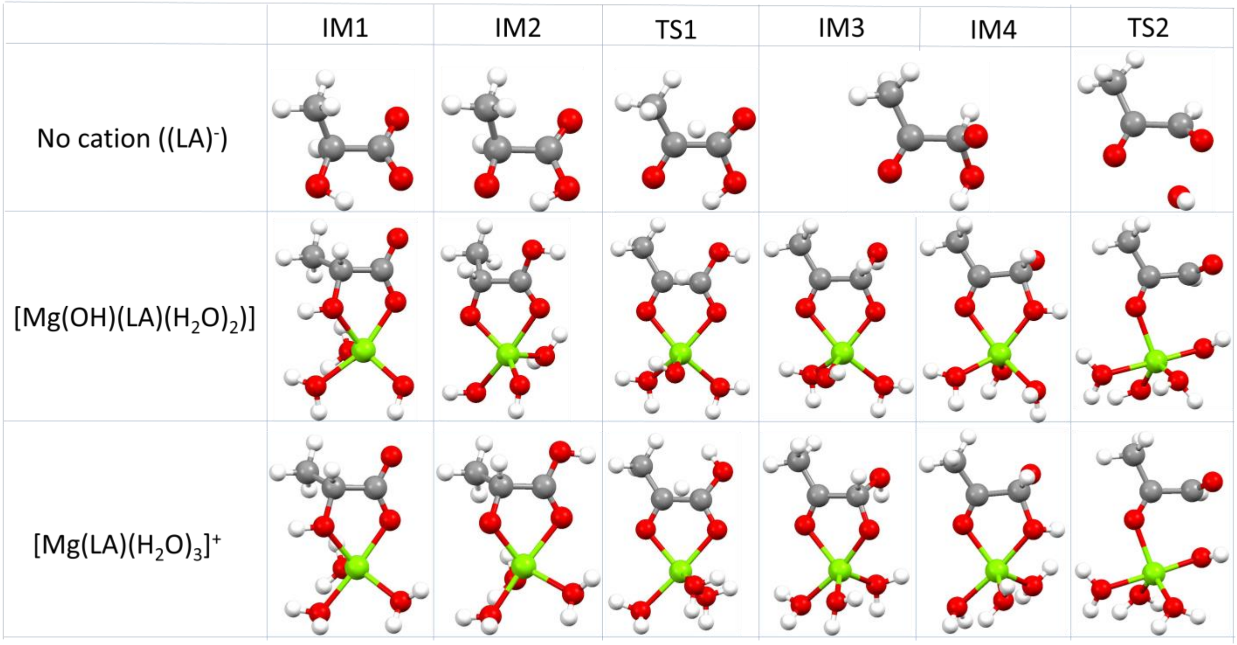

Figure S4. Optimised geometry of species involved including IM and TS.

References

1. M. Valiev, E. J. Bylaska, N. Govind, K. Kowalski, T. P. Straatsma, H. J. J. Van Dam, D. Wang, J. Nieplocha, E. Apra, T. L. Windus and W. de Jong, *Computer Physics Communications*, 2010, **181**, 1477-1489.
2. S. Kozuch and S. Shaik, *Accounts Chem. Res.*, 2011, **44**, 101-110.
